# Improving confidence intervals for normed test scores: Include uncertainty due to sampling variability

**DOI:** 10.3758/s13428-018-1122-8

**Published:** 2018-11-06

**Authors:** Lieke Voncken, Casper J. Albers, Marieke E. Timmerman

**Affiliations:** Department Psychometrics & Statistics, Faculty of Behavioural and Social Sciences, University of Groningen, Grote Kruisstraat 2/1, 9712 TS Groningen, The Netherlands

**Keywords:** Continuous norming, GAMLSS, Box-Cox power exponential distribution, Posterior simulation, Psychological tests

## Abstract

Test publishers usually provide confidence intervals (CIs) for normed test scores that reflect the uncertainty due to the unreliability of the tests. The uncertainty due to sampling variability in the norming phase is ignored. To express uncertainty due to norming, we propose a flexible method that is applicable in continuous norming and allows for a variety of score distributions, using Generalized Additive Models for Location, Scale, and Shape (GAMLSS; Rigby & Stasinopoulos, 2005). We assessed the performance of this method in a simulation study, by examining the quality of the resulting CIs. We varied the population model, procedure of estimating the CI, confidence level, sample size, value of the predictor, extremity of the test score, and type of variance-covariance matrix. The results showed that good quality of the CIs could be achieved in most conditions. The method is illustrated using normative data of the SON-R 6-40 test. We recommend test developers to use this approach to arrive at CIs, and thus properly express the uncertainty due to norm sampling fluctuations, in the context of continuous norming. Adopting this approach will help (e.g., clinical) practitioners to obtain a fair picture of the person assessed.

**Electronic supplementary material:**

The online version of this article (10.3758/s13428-018-1122-8) contains supplementary material, which is available to authorized users.

## Introduction

Norms are needed to give an interpretation of someone’s test score. A normed score can be expressed in different ways, like a percentile and *z* score. It indicates the person’s relative standing on the test to other people in the population. For instance, the normed scores of intelligence tests are typically expressed as normalized intelligence quotient (IQ) scores, with a population mean of 100 and standard deviation of 15, yielding an immediate interpretation of any observed IQ score.

Normed tests are often applied as high-stakes tests, meaning that they are used to make important decisions about individuals. A clear example relates to the fact that mentally retarded individuals are exempted from death penalty in 18 of the United States (Death Penalty Information Center, 2015). Some states, like Idaho and Florida, use IQ scores to identify mental retardation, applying a rigid cutoff (i.e., observed IQ score ≤ 70). Another instance of the use of a rigid cutoff can be found in the Netherlands, where mental retardation indicated by an observed IQ score of 85 or below qualifies for the long-term care act (Zorginstituut Nederland, 2017), allowing the financing of supervised living and debt repayment programs.

In using test scores for important individual decisions, it is essential to acknowledge the uncertainty in observed test scores. There is an increasing awareness of the importance of reflecting this uncertainty. For instance, in the fifth edition of the DSM (Diagnostic and Statistical Manual of Mental Disorders; American Psychiatric Association, 2013), unlike earlier editions, a standard error of 5 IQ points was explicitly included in defining the upper range of intellectual disability. These expressions of uncertainty in observed test scores reflect the notion that observed scores may differ across assessments, even if the individual assessed would remain exactly the same, or two individuals would be exactly the same, on the characteristic measured.

In line with this increased awareness, the Dutch Committee on Testing (COTAN) recommends test publishers to report information regarding the accuracy of the test (i.e., standard error of measurement, standard error of estimate, or test information function/standard error) and the appropriate intervals (Evers et al., 2009). Nowadays, many test publishers express this uncertainty related to test reliability, e.g. the WISC-IV (Wechsler, 2003) and the Bayley-III (Bayley, 2006).

Nevertheless, this is insufficient for normed scores, because it ignores another source of uncertainty, namely due to the test norming itself. Test norming takes place on the basis of a norming sample, rather than the full population, implying that the norms themselves are due to sampling fluctuations. This source of uncertainty in normed test scores has been acknowledged only recently, with the proposal of two methods to estimate CIs for normed test scores, under the assumption that the norming sample stems from a single population.

Crawford et al., (2011) proposed a method to obtain CIs around percentile norms, under the assumption that the scores in the norm population are normally distributed. Recently, Oosterhuis et al., (2017) derived standard errors for four different norm statistics (standard deviation, percentile ranks, stanine boundaries, and *z* scores), under the assumption that the scores in the norm population stem from a multinomial distribution. As described by Oosterhuis et al., (2016), this method can be applied to residuals of raw test scores in the context of regression-based norming, in which relevant personal characteristics (e.g., age) are used to estimate the raw test score distribution. Even though the method of Oosterhuis et al., (2017) has less strict assumptions than the method of Crawford et al., (2011), it still assumes normally distributed errors and homoscedasticity of the error variances, which are often unrealistic assumptions in practice. For instance, floor- and ceiling effects may introduce skewness.

We propose a method to derive CIs indicating uncertainty in normed scores that does not rely on those strict assumptions. To this end, we use the flexible Generalized Additive Models for Location, Scale, and Shape (GAMLSS; Rigby and Stasinopoulos, 2005), which has been advocated as a useful approach to continuous norming (e.g., Bayley-III (Bayley, 2006) and SON-R 2-8 (Tellegen & Laros, 2017)). GAMLSS includes a broad range of distributions, yielding a good chance of finding a well-fitting distribution for empirical normative data. Interestingly, the ordinary linear regression model described by Oosterhuis et al., (2016) is a restricted, special case of a model within the GAMLSS framework.

## GAMLSS

Applying GAMLSS implies that the score distribution is modelled conditional on predictor(s) of interest (e.g., age), based on certain distributional parameters. For instance, the Box-Cox power exponential (BCPE; Rigby and Stasinopoulos, 2004) distribution is a flexible continuous distribution, involving four distributional parameters, which relate to the location (*μ*), scale (σ), skewness (ν), and kurtosis (τ). These distributional parameters can be estimated as a function of predictor(s), like age. Once a model (e.g., μ = β_0_ + β_1_age + β_2_age^2^) has been selected for each of these distributional parameters, this estimated relationship between the predictor(s) and the distributional parameters fully determines the distribution of the test scores given the scores on the predictor(s). This distribution can then be used to calculate for any given testee what the relative position (i.e., normed score) of his/her observed score is within the estimated conditional score distribution.

So, if the only predictor is age, the normed scores can be determined for every possible test score conditional on every age value within the age range of interest. In this study, we focus on GAMLSS models with the BCPE distribution. We presume that a proper fitting BCPE model can be selected for the normative data at hand. The automated model selection procedure (Voncken et al., 2017) has been shown to perform well in the context of continuous norming. Note that extensive model fitting, followed by norming based on the same data, might lead to some overfitting.

Further, we focus on norms in the form of percentiles. This does not limit its applicability, because CIs of one type of norm statistic can easily be transformed to CIs of any other type of norm statistic (e.g., IQ scores, *z* scores, stanines). Hence, it is not necessary to derive the CI for every norm statistic separately. For instance, percentiles can be transformed to (normalized) *z* scores with the inverse normal distribution. A percentile of 50 is equal to a *z* score of 0. The *z* score can be transformed to an IQ score by multiplying the *z* score by the standard deviation of the desired distribution (i.e., 15), and then adding the mean of the distribution (i.e., 100).

### Estimating CIs for percentiles

#### **Posterior simulation procedure**

Once the BCPE model has been selected for the normative data at hand, the point estimates for the percentiles, conditional upon the predictor(s) (e.g., age), can be readily obtained as a quantity derived from the fitted model. To make inferences about quantities derived from a fitted GAMLSS model, the recommended method is posterior simulation (Wood, 2006). With this method, the parameter estimates are simulated conditional on the data.

In our study, we denote the CI that captures sampling fluctuation as CI_norm_, to explicitly distinguish from the CI that captures test unreliability, denoted as CI_rel_. By denoting the normed scores (e.g., percentiles, IQ scores) as 𝜃_norm_, we define CI_norm_ as the CI for 𝜃_norm_, thus capturing the uncertainty in the normed scores due to sampling variability. We propose to estimate CI_norm_ with a posterior simulation procedure consisting of the six steps described below. Table 1 provides an overview of the notation within the posterior simulation procedure.

1. Select a continuous norming model (e.g., with the automated model selection procedure described before).
2. Estimate the model parameters, denoted by $\hat {\uptheta }_{\text {par}}$, of the continuous norming model, and their covariances. The estimated model parameters, $\hat {\uptheta }_{\text {par}}$, involve all estimated parameters for each of the four distributional parameters of the BCPE distribution (i.e., $\boldsymbol {\hat {\upbeta }_{\upmu }}, \boldsymbol {\hat {\upbeta }_{\upsigma }}, \boldsymbol {\hat {\upbeta }_{\upnu }}$, and $\boldsymbol {\hat {\upbeta }_{\uptau }}$). For example, if the model for each distributional parameter involves a linear effect of one predictor, there are 8 estimated model parameters: 4 intercepts and 4 linear terms of the predictor.
3. Simulate $\hat {\uptheta }^{\textbf {s}}_{\text {par}}$, from a multivariate normal distribution: $\hat {\uptheta }^{\textbf {s}}_{\text {par}} \sim \mathbf {\mathcal {N}}\left (\hat {\uptheta }_{\text {par}}, {\Sigma }(\hat {\uptheta }_{\text {par}})\right )$, where $\hat {\uptheta }_{\text {par}}$ represents the vector of the parameter estimates, and ${\Sigma }(\hat {\uptheta }_{\text {par}})$ represents the corresponding estimated variance-covariance matrix.
4. Compute from the model with $\hat {\uptheta }^{\textbf {s}}_{\text {par}}$ the estimated normed scores of interest, $\hat {\uptheta }^{\textbf {s}}_{\text {norm}}$, for the test taker’s test score conditional on the predictor value(s) (e.g., test taker’s age) of interest. Repeat steps (3) and (4) many (e.g., *S* = 5,000) times.
5. Construct a distribution, $\hat {\uptheta }^{\textbf {s*}}_{\text {norm}}$, of the *S* estimated normed scores for the test taker $\hat {\uptheta }^{\textbf {s}}_{\text {norm}}$ computed in this process. This distribution contains the estimated normed scores of interest corresponding to each of the *S* sets of simulated model parameters.
6. Estimate CI_norm_ based on the constructed distribution, using the percentiles or the standard deviation of the distribution, depending on the method of estimating the CI.

**Table 1:** Notation within the posterior simulation procedure

Parameter	Definition
𝜃_par_	Set of model parameters of the continuous norming model in the population. This involves all parameters for each of the distributional parameters (e.g., **β**_**μ**_,**β**_**σ**_,**β**_**ν**_, and **β**_**τ**_ for the BCPE distribution).
$\hat {\uptheta }_{\text {par}}$	Estimates of 𝜃_par_ based on the normative sample.
${\Sigma }(\hat {\uptheta }_{\text {par}})$	Variance-covariance matrix of $\hat {\uptheta }_{\text {par}}$.
$\hat {\uptheta }^{\textbf {s}}_{\text {par}}$	Simulated set of model parameters within the posterior simulation procedure, drawn from a multivariate normal distribution defined by $\hat {\uptheta }_{\text {par}}$ and ${\Sigma }(\hat {\uptheta }_{\text {par}})$.
𝜃_norm_	Normed scores (person parameters) under the population model with parameters 𝜃_par_.
$\hat {\uptheta }_{\text {norm}}$	Estimates of 𝜃_norm_ under the estimated model with parameters $\hat {\uptheta }_{\text {par}}$.
$\hat {\uptheta }^{\textbf {s}}_{\text {norm}}$	Estimated normed scores under the model with a simulated set of model parameters, $\hat {\uptheta }^{\textbf {s}}_{\text {par}}$, within the posterior simulation procedure.

Step (6) of our procedure involves the estimation of CI_norm_ from the constructed distribution $\hat {\uptheta }^{\textbf {s*}}_{\text {norm}}$. We will consider three methods to do this: Wald method, percentile method, and bias-corrected (BC) percentile method.

#### **Wald method**

The Wald CI_norm_ is based on $\hat {\uptheta }_{\text {norm}}$ and the standard error SE^∗^, the standard deviation of the distribution. The lower and upper bounds of the 100(1-α)% CI are given by $\hat {\uptheta }_{\text {norm}} - {z}^{(\frac {1}{2}\upalpha )} \cdot \text {SE}^{*}$ and $\hat {\uptheta }_{\text {norm}} + {z}^{(1-\frac {1}{2}\upalpha )} \cdot \text {SE}^{*}$, respectively, where α is the significance level and *z*^(α)^ the 100αth percentile from the standard normal distribution.

#### **Percentile method**

The percentile CI_norm_ is based on the 100($\frac {1}{2}\upalpha $)th and 100(1-$\frac {1}{2}\upalpha $)th percentile of the cumulative distribution. The lower and upper bounds of the 100(1-α)% CI_norm_ are given by $\hat {\uptheta }_{\text {norm}}^{\textbf {s*}(\frac {1}{2}\upalpha )}$ and $\hat {\uptheta }_{\text {norm}}^{\textbf {s*}(1-\frac {1}{2}\upalpha )}$, respectively, where $\hat {\uptheta }_{\text {norm}}^{\textbf {s*}(\upalpha )}$ reflects the 100αth percentile of $\hat {\uptheta }_{\text {norm}}^{\textbf {s*}}$.

#### **Bias-corrected percentile method**

The bias-corrected percentile method (BC; Efron, 1982 p. 82) corrects the percentiles of the distribution for bias (i.e., the discrepancy between the centre of distributions $\hat {\uptheta }_{\text {norm}}^{\textbf {s*}}$ and $\hat {\uptheta }_{\text {norm}}$). The BC method estimates the lower and upper bounds of the 100(1-α)% CI_norm_ by $\hat {\uptheta }_{\text {norm}}^{\textbf {s*}({\upalpha }_{1})}$ and $\hat {\uptheta }_{\text {norm}}^{\textbf {s*}({\upalpha }_{2})}$, respectively, where α_1_ and α_2_ are estimated as

1 $$ \begin{array}{ll} \alpha_{1} =& \mathbf{\Phi} \left( 2\hat{z}_{0} + z^{(\frac{1}{2}\upalpha)} \right) \\ \alpha_{2} =& \mathbf{\Phi} \left( 2\hat{z}_{0} + z^{(1-\frac{1}{2}\upalpha)} \right). \end{array} $$

Φ(⋅) is the standard normal cumulative distribution function. The bias correction, $\hat {z}_{0}$, is equal to the proportion of estimators $\hat {\uptheta }^{\textbf {s}}_{\text {norm}}$ smaller than the sample estimate $\hat {\uptheta }_{\text {norm}}$,

2 $$ \begin{array}{ll} \hat{z}_{0} = \mathbf{\Phi}^{-1} \left( \frac{\# (\hat{\uptheta}_{\text{norm}}^{\textbf{s*}} < \hat{\uptheta}_{\text{norm}} )}{S}\right), \end{array} $$

where Φ^− 1^(⋅) is the inverse of Φ(⋅), and *#* is the count function. The BC method reduces to the percentile method if $\hat {z}_{0}$ equals 0.

The variance-covariance matrix of the parameter estimates, ${\Sigma }(\hat {\uptheta }_{\text {par}})$, which is required in step (3), may be estimated unreliably in case of additive terms (e.g., polynomials) and/or link functions other than the identity link (e.g., log link) (Stasinopoulos et al., 2015). As most distributions within the GAMLSS framework use link functions other than the identity link and additive terms are typically required to obtain good fit, it is not guaranteed that proper CIs follow from this procedure. To assess to what extent and under which conditions the posterior simulation procedure yields proper CI_norm_s for the normed score estimates, we performed a simulation study.

### Research Questions

The goal of this study is to assess the quality of the estimated CI_norm_s derived by our posterior simulation procedure, using two different population models (based on the SON-R 6-40 and FEEST normative data), three different CI methods (Wald, percentile, and bias-corrected percentile), two different confidence levels (CI90 and CI95), three different sample sizes (*N* = 501; 1,001; and 2,001), and two different methods of estimating the variance-covariance matrix ${\Sigma }(\hat {\uptheta }_{\text {par}})$. The CI_norm_s will be determined for all combinations of four different age values and three different true percentiles (5, 50, and 95). The quality of the CI_norm_s will be assessed in terms of coverage (i.e., proportion of CI_norm_s that cover the population parameter). Additionally, we investigate the proportion of CI_norm_s that missed the true score on the left or right side of the CI_norm_, and the length of the CIs. In general, there is a trade-off between the coverage and the CI length (Frangos & Schucany, 1990).

Theoretically, the percentile methods are preferred over the Wald method. Unlike the percentile methods, the Wald method is neither transformation respecting (i.e., CI changes with transformations) nor range preserving (i.e., the CIs can fall outside the allowable range of the statistics) (Efron & Tibshirani, 1993 pp. 174-175). In addition, the BC percentile method is preferred over the percentile method, as the former corrects for bias (i.e., asymmetry in the distribution). That is why we expect the BC percentile method to outperform the percentile method, and the percentile method to outperform the Wald method.

We expect the coverage to be better for the 90% CI_norm_ than for the 95% CI_norm_ because the latter requires more information about the tails of the distribution, which are difficult to estimate (Efron & Tibshirani, 1993 e.g., p. 275). Moreover, we expect an increase in sample size to result in smaller CI_norm_s.

We use two different methods to estimate ${\Sigma }(\hat {\uptheta }_{\text {par}})$: a standard variance-covariance matrix (‘vcov’) and a robust variance-covariance matrix (‘rvcov’), which has somewhat larger SEs. In general, the robust version is more reliable than the standard version when the variance model is suspected not to be correct, given that the mean model is correct (see Stasinopoulos et al., 2015 for more details). Given that we use the same mean estimates for the standard and robust variance-covariance matrix, we expect the coverage to be better for the latter. However, this also means that the CI_norm_ of the robust version is larger than the standard version.

We expect the coverage to be better for mid-range age values compared to age values at the extremes, as in the middle of the age range more information is available from surrounding age values to estimate the normed scores. Moreover, as more observations are present around the scores corresponding to the 50th percentile than scores corresponding to the 5th and 95th percentile of the conditional score distribution, we expect better coverage for the 50th percentile than the 5th and 95th percentile. This is in line with the findings of Oosterhuis et al., (2017), who concluded that extreme percentile ranks had poor coverage of CI_norm_s for small sample sizes (*N* < 1,000). Finally, we expect the CI_norm_s for the 50th percentile to be wider than those of the 5th and 95th percentiles.

## Method

**Fig. 1:**
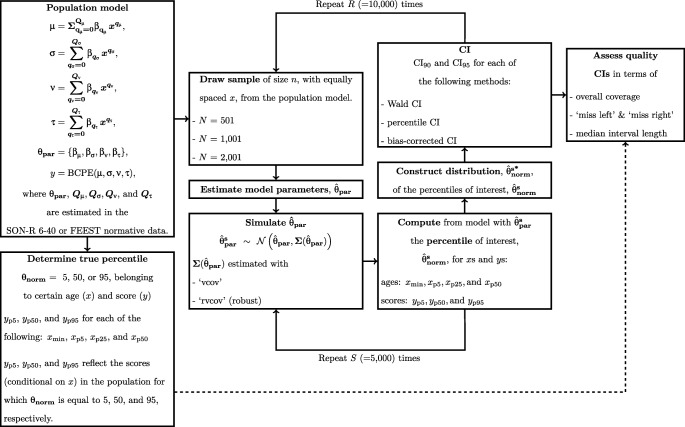
Schematic overview of the simulation study. A solid arrow indicates a next step in the procedure. A dotted arrow indicates the use of a result for comparison

The various conditions and different steps in the simulation study will be explained now. A schematic overview is presented in Fig. 1. The R code that was used for the simulation study and the analyses can be found on the Open Science Framework (OSF) via http://osf.io/z62xm/?view_only=8af3a8c83d76496a8651964f25835736.

### Population models

In this paper, we studied two population models: the estimated norming model of the SON-R 6-40 (Tellegen and Laros, 2014), which is a non-verbal intelligence test, and the estimated norming model of the Ekman 60 faces test, which is a facial emotion recognition test part of the Facial Expressions of Emotion – Stimuli and Tests (FEEST; Young et al., 2002; Voncken et al., 2018). The BCPE distribution (Rigby & Stasinopoulos, 2004) within the GAMLSS framework (Rigby & Stasinopoulos, 2005) is used to model the score distribution as a function of predictors for each of the four distributional parameters (μ, σ, ν, and τ). The SON-R 6-40 has one predictor, which is age, and the FEEST has three predictors: age, sex, and education. The model selection of the SON-R 6-40 test was done with the ‘free order’ automated model selection procedure in combination with the BIC as selection criterion (Voncken et al., 2017). As this ‘free order’ automated model selection procedure is not yet developed for multiple predictors, the model selection of the FEEST was done with a combination of the BIC and visual checks (i.e., worm plots; van Buuren & Fredriks, 2001). The population model parameters can be found in the Appendix.

### Conditions

#### **True percentile**

The true percentile, 𝜃_norm_, was equal to 5, 50 or 95. We determined the true scores *y* corresponding to those percentiles, conditional on the age values *x* of interest. For the FEEST model, sex and education were fixed to females and education category 6 (‘finished higher secondary education and/or college (not university)’), respectively. As we wanted to examine extreme age values and values closer to the middle of the age range (6 ≤ *x* ≤ 41 for SON-R 6-40 and 16 ≤ *x* ≤ 92 for FEEST), we investigate *x*_min_, *x*_p5_, *x*_p25_, and *x*_p50_, which corresponds to age values of 6, 7.75, 14.75, and 23.5 (SON) and 16, 19.8, 35, and 54 (FEEST). We investigate only one half of the age range, as the other half includes similar extremities. Given age, we investigated the score *y* in the population for which 𝜃_norm_ was equal to 5, 50, or 95.

#### **Sample size**

New data were generated for each different sample size. The sample sizes (*N*) are equal to 501, 1,001, and 2,001. These sample sizes are in the typical range of what is being used in practice. The age values *x* in each sample were fixed to be *N* equally spread values ranging from 6 to 41 (SON-R 6-40) or 16 to 92 (FEEST), as in the empirical data. The sample sizes are not rounded to hundreds to avoid age values with many decimal places.

#### **Type of**${\Sigma }(\hat {\uptheta }_{\text {par}})$

Within each data set, we varied whether ${\Sigma }(\hat {\uptheta }_{\text {par}})$ is equal to the standard variance-covariance matrix or the robust variance-covariance matrix, as provided by the software.

#### **CI method**

For each data set, we constructed the CI_norm_s using the Wald, percentile, and bias-corrected percentile methods. When the Wald method was used, we applied a logit transformation to the percentile distribution $\hat {\uptheta }^{\textbf {s*}}_{\text {norm}}$ before calculating SE^∗^. The rationale for this is that the range of percentiles is restricted to the range 0 − 100 (0 − 1 in proportions). Afterwards, the inverse logit transformation was applied to get the percentiles corresponding to the lower and upper bounds of the CI.

#### **Confidence level**

For each data set, we varied the confidence level, and constructed a 90% CI_norm_ (CI90) and a 95% CI_norm_ (CI95).

#### **Number of replications***R*

Every replication of the posterior simulation procedure resulted in a single CI_norm_. To assess the coverage of this CI, we replicated this procedure many times: That is, the number of replicated data sets per condition was fixed to *R* = 10,000. This number was chosen to ensure a maximal width CI95 of the coverage estimates themselves of 0.02. The coverage estimate follows a binomial distribution because each individual CI_norm_ does either contain the true value, with expected probability *p* equal to 1 −α, or does not contain the true value, with expected probability equal to (1 − *p*), and is repeated *R* times. The variance of the proportion of CIs containing the true value is equal to $\frac {1}{R} p (1-p)$. The variance is largest when *p* = 0.50. The size of the CI95 corresponding to this maximum variance is equal to

3 $$ \begin{array}{ll} \text{CI95}_{\text{size}} & = 2 z_{1 - \upalpha/2} \sqrt{\textstyle\frac{1}{R} p \cdot (1-p)} \\ & = 2 z_{1 - \upalpha/2} \sqrt{\textstyle\frac{1}{R} 0.50 (1-0.50)} \\ & = 0.02, \end{array} $$

where *z*_1−α/2_ is the 1 −α/2 quantile of the standard normal distribution, equal to about 1.96. Then it follows that *R* should be equal to at least 9,603 in order to have a maximum CI95 size of 0.02, which we rounded up to *R* = 10,000. As the variance-covariance matrix was not always positive definite, we sampled from the population model until we got 10,000 results with a positive definite matrix.

#### **Number of simulations***S*

Step (3) of the posterior simulation procedure consists of simulating $\hat {\uptheta }_{\text {par}}$ from a multivariate normal distribution. The number of simulations *S* was fixed to 5,000. The larger *S*, the higher the precision of the estimated distribution. According to Efron and Tibshirani (1993, p. 52), *S* equal to 200 is usually more than enough when obtaining standard errors. However, *S* needs to be much larger when obtaining confidence intervals. In order to determine the required size of *S*, we calculated the lower- and upperbound of the CI_norm_s of percentile estimates for *S* ranging from 1,000 to 11,000, and one replication *r*. We fixed the sample size *N* to 501 and we used the standard variance-covariance matrix. We investigated the results for the three CI methods, two confidence levels, and twelve combinations of age and test scores. All results seemed to have converged after 11,000 simulations. We aimed at optimally balancing the trade-off between the desired precision and the computation time, by selecting the minimal number of simulations such that the maximum difference in estimated percentile between fewer simulations and the estimate of convergence was 0.5 percentile. This criterion was met for *S* = 5,000.

### Quality assessment

We assessed the quality of the estimated CI_norm_s in three different ways, ordered in terms of importance. First, we investigated the coverage, which is defined as the proportion of CI_norm_s containing the true percentile 𝜃_norm_. Ideal coverage means that this proportion is equal to 1 −α. Second, we investigated the proportion of CI_norm_s that missed the true percentile on the left (‘miss left’) or right (‘miss right’) side. For instance, if the true percentile is 50, miss left means that the left endpoint was above 50. The total of ‘miss left’ and ‘miss right’ can be calculated as 1 minus the coverage. Ideally, the values of miss left and miss right are both equal to α/2. Our outcome measure was the ratio ‘miss left’ to ‘miss right’. A ratio of 1 indicates that both proportions are equal, a ratio larger than 1 indicates that ‘miss left’ is larger than ‘miss right’, and a ratio smaller than 1 indicates that ‘miss right’ is larger than ‘miss left’. Third, we investigated the median interval length: the median absolute difference of the lower- and upper bound of the CI_norm_ over all replications *R*. Ideally, the CI_norm_ is small (i.e., precise), given that the coverage is good. A median interval length of 0.10 means that, over all 10,000 replications, the median width of the CI_norm_ was 10 percentile points.

Note that each outcome measure (coverage, ratio ‘miss left’ to ‘miss right’, and median interval length) is calculated across the 10,000 replications (e.g., proportion of replications for which CI_norm_ contains the true percentile). As a result, the total number of observations per outcome measure equals 3 (*N*) × 2 (${\Sigma }(\hat {\uptheta }_{\text {par}})$ method) × 2 (confidence level) × 3 (percentile) × 4 (age) = 144.

## Results

### Comparison CI methods in terms of coverage

To achieve an overview of the comparative performances of the CI methods, we first consider the coverage. Table 2 shows for the two population models the deviations between the ideal coverage (0.90 in the CI90 conditions and 0.95 in the CI95 conditions) and the observed coverage, averaged over the four age values and three percentiles, for the combinations of CI method, type of variance-covariance matrix, confidence level, and sample size. For example, a deviation of − 0.006 for CI95 means that the actual coverage, averaged over the four age values and three percentiles, was 0.944. In each row, per population model, the best performing method in terms of deviation from ideal coverage is bolded. We will discuss the results for the two population models separately.

**Table 2:** Deviation from ideal coverage, averaged over the 4 age values and 3 percentiles

		SON-R 6-40			FEEST		
*N* = 501		Wald	Percentile	Bias-corrected	Wald	Percentile	Bias-corrected
vcov	CI90	+ 0.020 (0.023)	**–0.016 (0.008)**	–0.025 (0.024)	+ 0.004 (0.010)	–0.010 (0.010)	**–0.001 (0.008)**
	CI95	**+ 0.012 (0.013)**	–0.015 (0.006)	–0.019 (0.014)	**–0.001 (0.007)**	–0.011 (0.010)	–0.012 (0.007)
rvcov	CI90	+ 0.022 (0.017)	**–0.010 (0.012)**	–0.019 (0.026)	+ 0.016 (0.003)	+ 0.007 (0.009)	**+ 0.005 (0.008)**
	CI95	**+ 0.011 (0.010)**	–0.014 (0.010)	–0.017 (0.017)	+ 0.009 (0.004)	**+ 0.001 (0.005)**	+ 0.002 (0.005)
*N* = 1,001		Wald	Percentile	Bias-corrected	Wald	Percentile	Bias-corrected
vcov	CI90	+ 0.013 (0.017)	**–0.006 (0.003)**	–0.015 (0.016)	+ 0.009 (0.007)	**⋆ (0.006)**	**⋆ (0.005)**
	CI95	+ 0.008 (0.011)	**–0.007 (0.003)**	–0.011 (0.009)	+ 0.004 (0.005)	**–0.002 (0.005)**	**–0.002 (0.003)**
rvcov	CI90	+ 0.016 (0.014)	**–0.002 (0.006)**	–0.011 (0.018)	+ 0.016 (0.003)	+ 0.007 (0.008)	**+ 0.006 (0.007)**
	CI95	+ 0.008 (0.009)	**–0.006 (0.005)**	–0.010 (0.011)	+ 0.008 (0.003)	**+ 0.001 (0.005)**	+ 0.002 (0.005)
*N* = 2,001		Wald	Percentile	Bias-corrected	Wald	Percentile	Bias-corrected
vcov	CI90	+ 0.008 (0.014)	**–0.001 (0.008)**	–0.008 (0.009)	+ 0.005 (0.004)	**+ 0.001 (0.004)**	**+ 0.001 (0.003)**
	CI95	+ 0.004 (0.009)	**–0.002 (0.004)**	–0.006 (0.005)	+ 0.003 (0.003)	**⋆ (0.003)**	**⋆ (0.003)**
rvcov	CI90	+ 0.010 (0.014)	**+ 0.001 (0.007)**	–0.007 (0.008)	+ 0.013 (0.004)	**+ 0.008 (0.005)**	**+ 0.008 (0.005)**
	CI95	+ 0.005 (0.008)	**–0.002 (0.004)**	–0.005 (0.004)	+ 0.008 (0.004)	**+ 0.004 (0.004)**	**+ 0.004 (0.005)**

Note SDs between parentheses. For each population model, the CI method with the smallest deviation from ideal coverage per row is bolded

⋆ Deviation between –0.001 and 0.001

#### **SON-R 6-40**

The results of the SON population model show that, in general, the coverage is close to the ideal coverage and the coverage becomes better as sample size increases. The standard deviation, which is given between parentheses, reflects the variation between the different age values and percentile conditions. The percentile method performs best in almost all conditions, in terms of both the mean deviation and its standard deviation. The percentile method is only outperformed by the Wald method when *N* = 501 and the confidence level equals .95. We indeed expected the percentile method to outperform the Wald method, but we didn’t expect the percentile method to outperform the bias-corrected percentile method as well.

#### **FEEST**

The results of the FEEST population model show that, in general, the coverage for this model is even closer to ideal coverage than in the SON population model. Again, coverage becomes better as sample size increases, but the increase is very small when going from *N* = 1,001 to 2,001, as the coverage is already very close to ideal coverage for *N* = 1,001. The percentile and bias-corrected methods perform about equally well, and they are only outperformed by the Wald method when *N* = 501, the confidence level equals .95, and the vcov method is used. This is in line with our expectations, as the percentile method didn’t outperform the bias-corrected method.

Tables S1, S2, and S3 of the supplementary material show the results separately for each of the three percentiles (i.e., 5, 50 and 95, respectively). The 5th and 95th percentiles are more interesting in the clinical and education context than the 50th percentile, because these contexts often involve a selection of the *x*% worst or best performing test takers. The results show that for the FEEST population model, the difference between the CI methods if small regardless of the percentile. For the SON population model, on the other hand, the difference between the CI methods are rather large for the 5th and 95th percentiles, and small for the 50th percentile. More specifically, for the 5th and 95th percentiles, the percentile CI method outperforms the Wald and bias-corrected CI methods in almost all conditions.

Taken together, the coverage of the FEEST population model is close to ideal coverage in almost all conditions. In contrast, the coverage of the SON population model varies depending on the different conditions. In addition, the percentile CI method performed well for both population models. That is why we will further investigate the effect of sample size, type of variance-covariance matrix, confidence level, percentile, and age for the SON population model and the percentile CI method only.

### Results SON population model and percentile CI method

To obtain insight into the effects of the factors on the absolute deviation from ideal coverage and the ratio ‘miss left’ to ‘miss right’ for the SON population model in combination with the percentile CI method, an analysis of variance (ANOVA) with main effects and 2-way interactions was performed. Higher order interactions were not taken into account because of interpretability issues. Note that we refrained from performing a mixed effects ANOVA to account for the within factors (i.e., type of variance-covariance matrix, confidence level, percentile, and age) because it is not possible to estimate the mixed effects ANOVA due to rank deficiency (since for each combination of the five factors only a single observation is available). Instead, we performed a between-subjects ANOVA. We believe ignoring the within structure, and ignoring ANOVA’s assumptions of normal and homoscedastic errors, is not problematic because we are interested in the relative effect of the factors rather than the exact results of the ANOVA. Table 3 shows the results from the ANOVA. We consider effects with partial ω^2^ < 0.2 for the deviation from ideal coverage and partial ω^2^ < 0.4 for ‘miss left’ to ‘miss right’ ratio to be too weak to study in more detail. We will describe the results for the median interval length only briefly, without tables or figures, as the coverage and ‘miss left’ to ‘miss right’ ratio are more important outcome measures.

**Table 3:** Partial ω^2^s of absolute deviation from ideal coverage and ratio ‘miss left’ to ‘miss right’ for the percentile CI method and the SON-R 6-40 population model

Source	Deviation	MLMR
*N*	**.564**	**.457**
$\boldsymbol {\Sigma }(\hat {\uptheta }_{\text {par}})$ method	⋆	−.007
confidence level	−.005	.169
percentile	**.243**	**.967**
age	.069	**.517**
*N* × $\boldsymbol {\Sigma }(\hat {\uptheta }_{\text {par}})$ method	−.008	−.012
*N* × confidence level	.017	.057
*N* × percentile	**.209**	**.697**
*N* × age	.060	.252
$\boldsymbol {\Sigma }(\hat {\uptheta }_{\text {par}})$ method × confidence level	−.001	−.007
$\boldsymbol {\Sigma }(\hat {\uptheta }_{\text {par}})$ method × percentile	.045	−.013
$\boldsymbol {\Sigma }(\hat {\uptheta }_{\text {par}})$ method × age	.061	−.018
confidence level × percentile	−.006	**.441**
confidence level × age	.016	.051
percentile × age	**.271**	**.672**

Note Deviation = absolute deviation from ideal coverage. MLMR = ratio ‘miss left’ to ‘miss right’. *N* = sample size. The effects of the SON-R 6-40 population model with partial ω^2^ ≥ .2 (Deviation) and partial ω^2^ ≥ .4 (MLMR), which we inspected more closely, are displayed in bold font. ⋆ Partial ω^2^ between –0.001 and 0.001

#### **Coverage**

The ANOVA results for the absolute deviation from ideal coverage are shown in Column ‘Deviation’. The main effects of *N* and percentile, and the interaction effects between *N* and percentile, and percentile and age have partial ω^2^ ≥ 0.2.

**Fig. 2:**
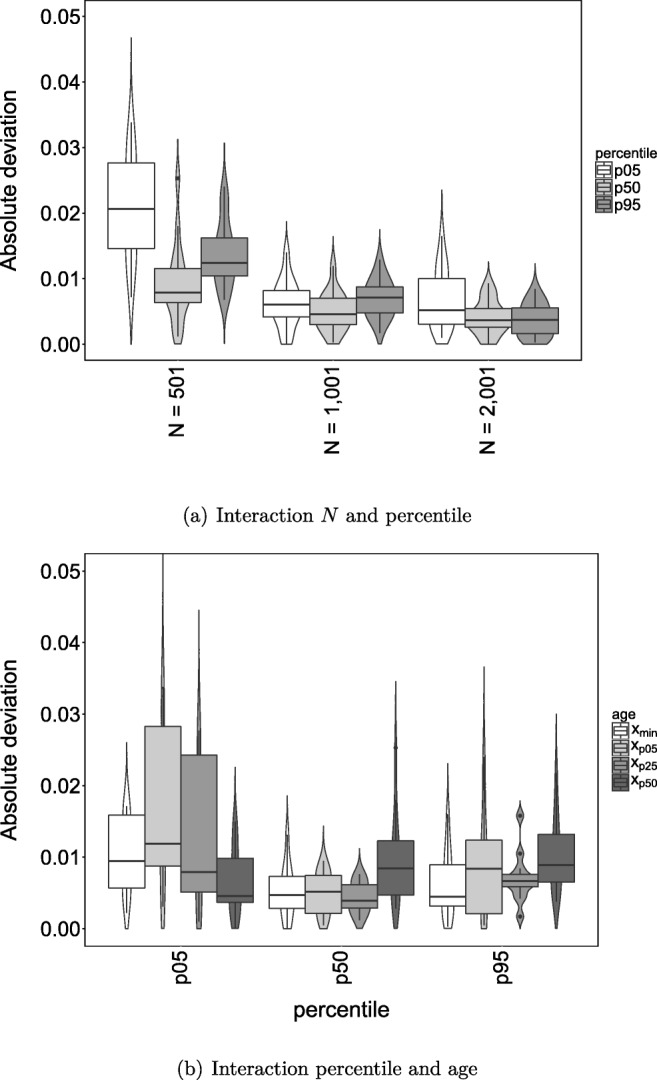
Violin plots with boxplots depicting the absolute deviation from ideal coverage with the percentile CI, for the interaction between *N* and percentile, and percentile and age

The main and interaction effects are shown in Fig. 2. Panel (a) shows the interaction effect between *N* and percentile. It shows that the mean absolute deviation from ideal coverage decreases with increasing sample size. We expected the coverage to be better for the 50th percentile than the 5th and 95th percentiles. This is indeed what we have found. This effect diminishes as sample size increases, as the absolute deviations of all three percentile conditions get closer to zero with increasing sample size.

Panel (b) shows the interaction effect between percentile and age. The effect of percentile is in general the same as in panel (a). We expected the coverage to be better for mid-ranged age values than more extreme age values. However, we did not always find that coverage was better for mid-ranged age values. For the 5th percentile, the absolute deviation from ideal coverage indeed becomes smaller as the age value becomes less extreme, except that *x*_p5_ has a slightly higher deviation compared to *x*_min_. For the 50th percentile, the four age values have a rather similar absolute deviation, but *x*_p50_ has the highest absolute deviation. For the 95th percentile, the differences in absolute deviation between the age values are larger again. The absolute deviation is largest for *x*_p5_ and *x*_p50_. Overall, the deviation from ideal coverage is quite small. A maximum absolute deviation of 0.01 means that the coverage of the 90% CI_norm_ was between 0.89 and 0.91.

Contrary to our expectations, there seems to be no effect of ${\Sigma }(\hat {\uptheta }_{\text {par}})$ method and confidence level on the absolute deviation from ideal coverage.

#### **Miss left and miss right**

**Fig. 3:**
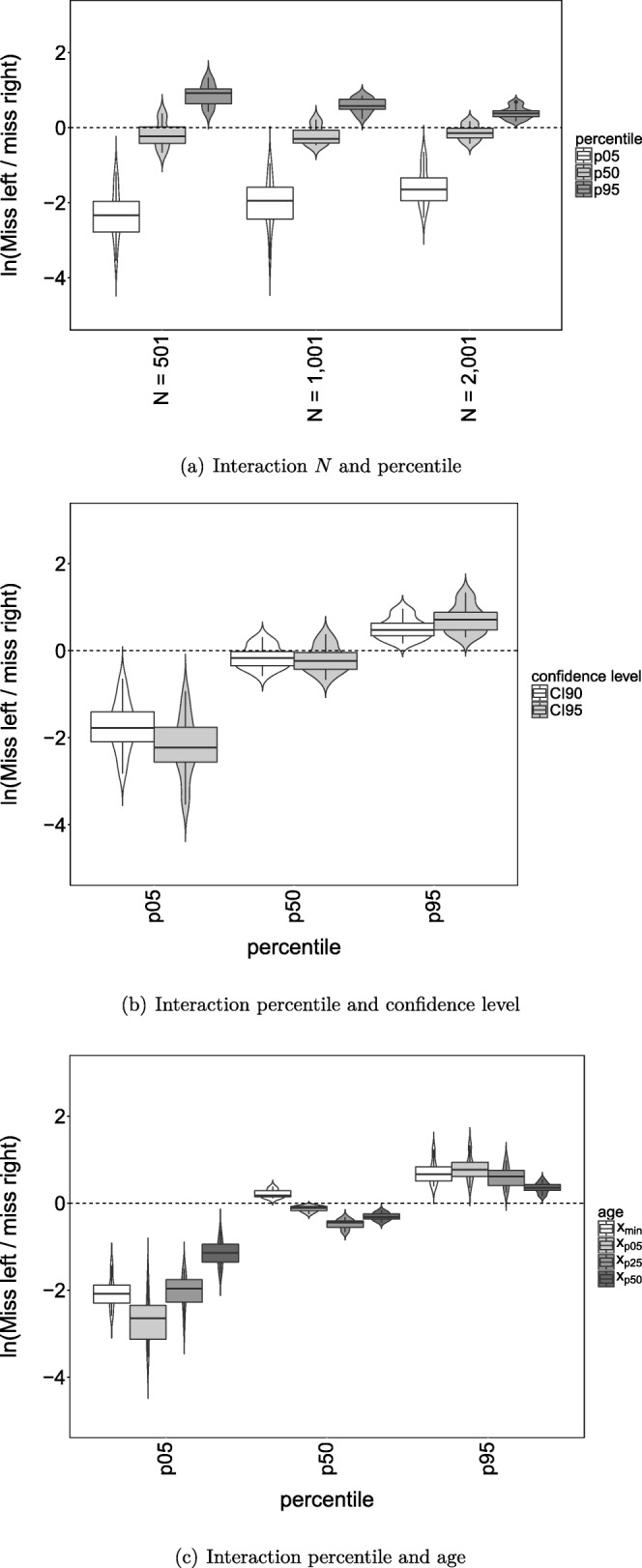
Violin plots with boxplots depicting the ‘miss left’ to ‘miss right’ ratio on a logarithmic scale with the percentile CI, for the interactions between *N* and percentile, confidence level and percentile, and percentile and age

The ANOVA results for the ratio ‘miss left’ to ‘miss right’ are shown in Column ‘MLMR’. The main effects of *N*, percentile and age, and the interaction effects between *N* and percentile, confidence level and percentile, and percentile and age have ω^2^ ≥ 0.4. These main and interaction effects are shown in Fig. 3. The dashed line represents the point where ‘miss left’ and ‘miss right’ are equal. The vertical axis shows the ‘miss left’ to ‘miss right’ ratio on a logarithmic scale. As a result, positive values on the *y* axis indicate that ‘miss left’ is larger than ‘miss right’, and negative values indicate that ‘miss right’ is larger than ‘miss left’. In addition, the absolute vertical distance from the dashed line (*y* = 0) represents the same effect size above and below the dashed line. That is, for instance, a value of 0.7 indicates that ‘miss right’ is about twice as large as ‘miss left’, and a value of − 0.7 indicates that ‘miss left’ is about twice as large as ‘miss right’.

Percentile is involved in all three interactions effects. Panel (a), (b), and (c) show that the log ratio of ‘miss left’ and ‘miss right’ is only about equal to zero for the 50th percentile. In general, ‘miss right’ is larger than ‘miss left’ for the 5th percentile and ‘miss left’ is larger than ‘miss right’ for the 95th percentile. In addition, it is shown that, regardless of percentile level, the log ratio becomes closer to zero as sample size increases (panel a) and is closer to zero for the 90% CI_norm_ compared to the 95% CI_norm_ (panel b). Finally, panel (c) shows that, for the 5th and 95th percentile, the log ratio is closest to zero for *x*_p50_, and, for the 50th percentile, *x*_min_ has a log ratio slightly above zero, while the other age values have a log ratio slightly below zero.

#### **Median interval length**

We expected the median interval length to be smaller as sample size increases, larger for the 50th percentile compared to the 5th and 95th percentiles, and larger for ‘rvcov’ than for ‘vcov’. The first two expectations are indeed confirmed, but there seems to be no effect of ${\Sigma }(\hat {\uptheta }_{\text {par}})$ method on the median interval length. For each of the 72 conditions considered, the ratio of the median interval lengths of the ‘rvcov’ and ‘vcov’ methods lied between 1.00 and 1.09. In addition, we found that the median interval length becomes smaller as age becomes less extreme.

### Discussion simulation study

Based on these results, we conclude that in most conditions the coverage of CI_norm_ is good. As we generally want to construct CI_norm_s across the whole range of the score distribution, for all possible age values, we recommend to use the percentile CI method, in combination with a large sample size (see Table 2). The percentile CI method especially outperforms the Wald and bias-corrected CI methods for the 5th percentile (see supplementary tables). The 95% CI_norm_ appears to be more difficult to estimate than the 90% CI_norm_, as the latter has more similar ‘miss left’ and ‘miss right’ values. We don’t have a preference for a ${\Sigma }(\hat {\uptheta }_{\text {par}})$ method, as we did not find a clear effect of this on our outcome measures.

### Empirical illustration construction CI_norm_

Using the ‘rvcov’ method for the variance-covariance matrix and the recommended percentile CI method, we illustrate with the SON-R 6-40 data (Tellegen & Laros, 2014) how to construct CI_norm_. The sample size of the SON-R 6-40 data is 1,933. This seems a reasonable sample size for our purposes, because in our simulation study that involved simulated data with a structure resembling these empirical data, a sample size close to 2,000 seemed sufficient to achieve proper estimates for CI_norm_.

The R code with the procedure to construct CI_norm_ for your own data can be found as supplemental material. This procedure allows you to construct CI_norm_ with a specified confidence level, for specified combinations of age and test score. The steps in this procedure are as follows: First, you have to load your data, specify the confidence level (e.g., CI95), and specify the combination(s) of age value and test score for which you want to calculate CI_norm_. Second, a model needs to be selected. We used the ‘free order’ automated model selection procedure (Voncken et al., 2017). This procedure selects the order of the orthogonal polynomials in each of the parameters related to the BCPE distribution (i.e., μ, σ, ν, and τ). With the chosen model, the parameter estimates and the corresponding variance-covariance matrix are obtained. Third, in the posterior simulation, 5,000 model parameters are simulated from a multivariate normal distribution, with the point estimates of the parameters as mean, and ‘rvcov’ as covariance matrix of the parameters. For each set of the 5,000 simulated model parameters, the corresponding percentiles are calculated for the specified combination(s) of age value and test score. Finally, based on the distribution(s) of the 5,000 resulting percentiles in the previous step, the confidence intervals are determined for each specified combination of age value and test score.

**Fig. 4:**
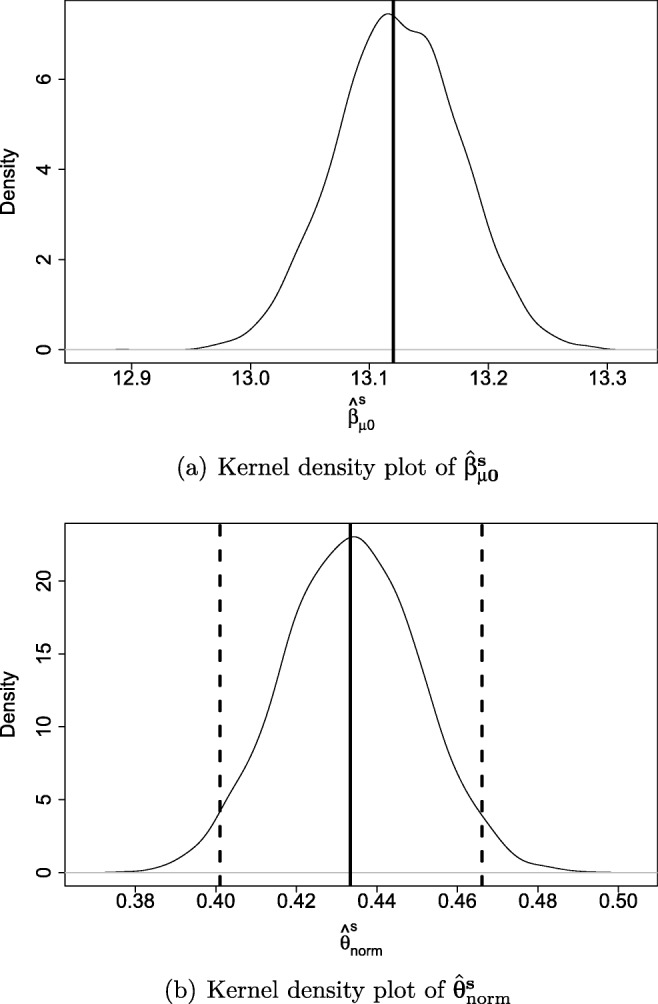
Kernel density plots illustrating the simulated distribution of the intercept parameter of µ, $\hat {\upbeta }_{\upmu \mathbf {0}}^{\textbf {s}}$, (panel a) and the distribution of percentiles, $\hat {\uptheta }_{\text {norm}}^{\textbf {s*}}$, corresponding to an age value of 8 and a test score of 9 (panel b). The vertical solid lines represent the point estimate (panel a) and the percentile corresponding to the point estimates of the distributional parameters (panel b), and the vertical dashed lines in panel b represent the bounds of the CI95_norm_

The last two steps of this procedure are illustrated in Fig. 4. In the third step, 5,000 model parameters are simulated. Panel (a) shows the simulated posterior distribution of the intercept term of distributional parameter μ, $\hat {\upbeta }_{\upmu \mathbf {0}}^{\textbf {s}}$. The vertical line represents the point estimate, $\hat {\upbeta }_{\upmu \mathbf {0}}$, which is the originally estimated model parameter in the second step. The distribution around it represents the 5,000 simulated intercept terms of distributional parameter μ.

Each estimated model parameter has its own simulated posterior distribution. For each set of the 5,000 simulated model parameters, the percentile for each combination of test score and age value was determined. That is, each of the 5,000 sets of simulated parameters resulted in a test score distribution conditional on age. Given a test taker’s age, the percentile corresponding to his/her test score can be derived for each of the 5,000 simulations.

Panel (b) shows the simulated posterior percentile distribution, $\hat {\uptheta }^{s*}_{\text {norm}}$, for a 8-year-old with a test score of 9. It shows the distribution of all 5,000 resulting percentiles, $\hat {\uptheta }_{\text {norm}}^{s}$. The vertical solid line represents the percentile corresponding to the point estimates of the estimated model parameters. The dashed vertical lines represent the bounds of the CI95_norm_ (2.5th and 97.5th percentiles of the distribution), which are equal to 40.1 and 46.6. The final step of the procedure involves determining those bounds of the CI based on the simulated posterior percentile distribution. So, using the percentile CI to derive CI95_norm_, the CI95_norm_ for a 8- year-old with a SON-R 6-40 test score of 9 is [40.1, 46.6].

#### **Comparison BCPE and normal distribution**

The chosen model here involved age dependence of the median, scale, skewness, and kurtosis of the score distribution, with polynomials of age up to degree four. This non-normality is very common for (psychological) normative data because of floor- and ceiling effects. In general, the disadvantage of a more flexible model (i.e., with more parameters to estimate) compared with a simpler model is that more observations are needed to estimate the model. However, if the assumptions of the simpler model are (strongly) violated, the improved model fit of the more complex model outweighs the costs of added complexity. When comparing a normal distribution (NO) with only age dependence of the mean (as in the BCPE model, the polynomial of degree 4 resulted in the best fit) and the BCPE distribution with age dependence of all four distributional characteristics, even with the double number of parameters (i.e., 12 vs. 6), the BCPE distribution model had a lower BIC value (i.e., 8655 vs. 8960) than the normal distribution model. This means that even when taking into account the number of parameters, the BCPE distribution model fits the normative data better than the normal distribution model.

**Fig. 5:**
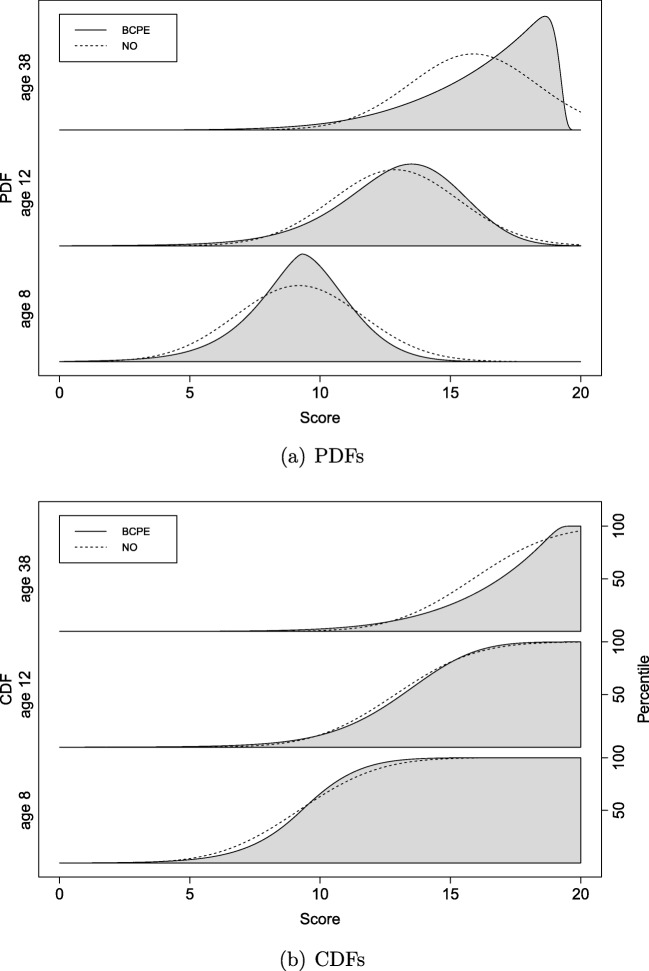
PDFs, panel **a**, and CDFs, panel **b**, for the SON-R 6-40 model estimated with the BCPE distribution (solid line) and normal distribution (NO; dashed line), conditional on three different age values (i.e., 8, 12, and 38-year-olds)

Figure 5 shows the estimated PDFs and CDFs conditional on three different age values (i.e., 8, 12, and 38-year-olds), for the BCPE distribution and normal distribution. The PDFs show the estimated conditional score distributions. For age 8, the BCPE and NO distributions are both (about) symmetric, but the BCPE distribution has a smaller variance and is leptokurtic. The older the test taker is, the larger the deviation from normality is. A clear ceiling effect is visible for older test takers, as indicated by strong negative skewness. The maximum obtained raw test score in the population was about 20. This is captured by the BCPE distribution, while the estimated normal distribution goes beyond this score for older test takers.

Importantly, because the norms are directly derived from the estimated conditional score distribution, the use of a bad fitting model directly affects the quality of the norm estimates. The CDFs show the percentile point estimates corresponding to the raw scores conditional on three age values. It may seem that the lines are relatively close to each other, but this is misleading. For instance, for the above described 8-year-old test taker with test score 9, the lines seem to be very close to each other, but the difference in percentile point estimate is 6.6. The corresponding CI95_norm_s are [40.1, 46.6] and [43.9, 49.9] for the BCPE and NO distribution, respectively. So, in this case, about half of the CI95_norm_s overlap. For older test takers, this difference in point estimates is even larger. For 38-year-old test takers with score 17, the difference in percentile point estimate is 16.2, with CI95_norm_s of [45.1, 57.2] and [60.1, 75.1] for the BCPE and NO distribution, respectively. Here, the CI95_norm_s have an overlap below 25%. This shows that not taking into account non-normality can greatly affect the estimated percentiles in empirical practice.

## Discussion

The results of the simulation study showed for two different population models, with one or three predictors, that the performance of the CI_norm_*s* was overall best for the percentile CI method. The application of the posterior simulation in combination with this method to construct CI_norm_ was illustrated for the SON-R 6-40 data. While a sample size of 2,001 resulted in the best performing CI_norm_*s*, the results showed that a sample size of 1,001 yielded only minor deteriorations in performance. For the FEEST population model, the difference in performance between those two sample sizes was even negligible. So, we conclude that a sample size of 1,001 is sufficient to achieve a reasonable precision for data with structures comparable to the ones of the simulated data.

### Practical implications

Oosterhuis et al., (2017) described how to link CI_norm_ and CI_rel_. They construct CI_rel_ around the individual test scores and CI_norm_ around the scores corresponding to the norm statistic (e.g., percentiles). Then, they use the heuristic rule that there is a significant difference between the two statistics if the overlap between the two CIs is 25% or less (Van Belle, 2003). This allows practitioners to check if a certain person has a test score above/below a certain norm value.

As an illustration, consider person *X* having a certain test score on an intelligence test, which corresponds to a point estimate of his/her IQ of 72 given his/her age. If this person’s IQ is at most equal to 70, the death penalty does not apply to this person. If we do not take into account any uncertainty, we conclude that person X’s IQ is higher than 70. However, there is some uncertainty around the normed test score due to test unreliability, which results in, for instance, CI_rel_ = [70, 74]. In addition, there is some uncertainty around the norms. Our bootstrap procedure provides you with the CI around the IQ of 70 given person X’s age, for instance, CI_norm_ = [68, 72]. As the overlap between the two CIs is larger than 25%, the IQ of person X does not differ significantly from the IQ_cutoff_ of 70. As a result, if we take into account both types of uncertainty, we conclude that the death penalty does not apply to person X.

### Limitations

This study has two possible limitations. First, we only used the BCPE distribution. Hence, we do not know the quality of the CIs for other distributions. The GAMLSS framework includes many other distributions, which might fit your data better. Fortunately, the BCPE distribution is applicable in many cases because of its flexibility. This distribution is generally suited for continuous outcome variables. For test score distributions that deviate substantially from a continuous distribution, GAMLSS may provide an alternative distribution, as for example the beta-binomial distribution for discrete numbers.

As we used both polynomials and the log link function for two distributional parameters (i.e., σ and τ), which might cause the variance-covariance matrix to be estimated unreliably, we do not expect the quality of the CIs to be worse for other distributions.

Second, as described in the method section, the variance-covariance matrix was not positive definite in some replications. For the SON data, for instance, the matrix was not positive definite in about 2.4% of the replications when *N* = 2,001, about 9.7% when *N* = 1,001, and about 25.4% when *N* = 501. This might be an indication that 501 (and 1,001) observations are not enough. We continued replicating until we had 10,000 results with positive definite matrices. In practice, you only have one replication, and it is possible that the matrix is not positive definite there. To deal with this, one could either use an algorithm to force positive definiteness (e.g., Knol and Ten Berge, 1989, Higham 2002), or tolerate a specified amount of lack of numerical positive-definiteness (in the procedure applied in the ‘mvrnorm’ function in the MASS package in R (Venables & Ripley, 2002)).

### General conclusion

We recommend test developers to use our approach to derive CI_norm_ because of its flexibility and because it is incorporated in the continuous norming process. It allows them to properly express the uncertainty due to norm sampling fluctuations. So, adopting this approach will help (e.g., clinical) practitioners to obtain a fair picture of the person assessed.

### Electronic supplementary material

Below is the link to the electronic supplementary material.

(PDF 110 KB)

(R 5.87 KB)

(TXT 39.7 KB)

## Appendix: Population model parameters

The population model parameters for distributional parameters μ (location), σ (scale), ν (skewness), and τ (kurtosis) are as follows

$$\begin{array}{ll} & \textbf{SON-R 6-40 model} \\ {\upmu}_{\text{SON}} & = {\upbeta}_{\upmu\boldsymbol{0}} + {\upbeta}_{\upmu\boldsymbol{1}} \cdot f_{1}(\text{age}) + {\upbeta}_{\upmu\boldsymbol{2}} \cdot f_{2}(\text{age}) + {\upbeta}_{\upmu\boldsymbol{3}} \cdot f_{3}(\text{age}) + {\upbeta}_{\upmu\boldsymbol{4}} \cdot f_{4}(\text{age}) \\ & = 13.12 + 102.80 \cdot f_{1}(\text{age}) -66.38 \cdot f_{2}(\text{age}) + 27.19 \cdot f_{3}(\text{age}) - 7.94 \cdot f_{4}(\text{age}), \\ {\upsigma}_{\text{SON}} & = {\upbeta}_{\upsigma\boldsymbol{0}} + {\upbeta}_{\upsigma\boldsymbol{1}} \cdot f_{1}(\text{age}) + {\upbeta}_{\upsigma\boldsymbol{2}} \cdot f_{2}(\text{age}) = -1.79 - 8.92 \cdot f_{1}(\text{age}) - 3.74 \cdot f_{2}(\text{age}), \\ {\upnu}_{\text{SON}} & = {\upbeta}_{\upnu\boldsymbol{0}} + {\upbeta}_{\upnu\boldsymbol{1}} \cdot f_{1}(\text{age}) = 2.44 + 44.61 \cdot f_{1}(\text{age}), \\ {\uptau}_{\text{SON}} & = {\upbeta}_{\uptau\boldsymbol{0}} + {\upbeta}_{\uptau\boldsymbol{1}} \cdot f_{1}(\text{age}) = 0.84 + 19.64 \cdot f_{1}(\text{age}), \\ & \textbf{FEEST model} \\ {\upmu}_{\text{FEEST}} & = {\upbeta}_{\upmu\boldsymbol{0}} + {\upbeta}_{\upmu\boldsymbol{1}} \cdot f_{1}(\text{age}) + {\upbeta}_{\upmu\boldsymbol{2}} \cdot f_{2}(\text{age}) + {\upbeta}_{\upmu\boldsymbol{3}} \cdot \text{sex}_{\text{female}} + {\upbeta}_{\upmu\boldsymbol{4}} \cdot \text{education}_{\text{6}} \\ & = 42.53 -23.02 \cdot f_{1}(\text{age}) - 18.80 \cdot f_{2}(\text{age}) + 0.90 \cdot \text{sex}_{\text{female}} + 4.92 \cdot \text{education}_{\text{6}}, \\ {\upsigma}_{\text{FEEST}} & = {\upbeta}_{\upsigma\boldsymbol{0}} = -1.59, \\ {\upnu}_{\text{FEEST}} & = {\upbeta}_{\upnu\boldsymbol{0}} + {\upbeta}_{\upnu\boldsymbol{1}} \cdot \text{age} + {\upbeta}_{\upnu\boldsymbol{2}} \cdot \text{education}_{\text{6}} = 9.04 - 0.08 \cdot \text{age} + 5.50 \cdot \text{education}_{\text{6}}, \\ {\uptau}_{\text{FEEST}} & = {\upbeta}_{\uptau\boldsymbol{0}} = 0.20, \end{array} $$

where *f*_*d*_(age) refers to an orthogonal polynomial of age, with degree *d*. The predictors sex and education level are fixed to females and education category 6, respectively.
