# Supplementary material for: Improving confidence intervals for normed test scores: Include uncertainty due to sampling variability
Source: Behav Res Methods. 2018 Nov 6;51(2):826–39. doi: 10.3758/s13428-018-1122-8 (PMC6478628; doi:10.3758/s13428-018-1122-8)
Supplement: Supplementary file 1 — (PDF 110 KB) [file 13428_2018_1122_MOESM1_ESM.pdf]

Table S1: Deviation from ideal coverage, averaged over the 4 age values, for the 5th percentile

		SON-R 6-40			FEEST		
N = 501		Wald	Percentile	Bias-corrected	Wald	Percentile	Bias-corrected
vcov	CI90	+0.039 (0.026)	**–0.022 (0.008)**	–0.054 (0.015)	–0.006 (0.007)	**–0.003 (0.010)**	–0.010 (0.005)
	CI95	+0.023 (0.014)	**–0.021 (0.006)**	–0.034 (0.010)	**–0.009 (0.006)**	**–0.009 (0.010)**	**–0.009 (0.005)**
rvcov	CI90	+0.035 (0.021)	**–0.019 (0.012)**	–0.047 (0.014)	+0.013 (0.003)	**+0.007 (0.009)**	**+0.007 (0.008)**
	CI95	**+0.020 (0.012)**	–0.021 (0.010)	–0.034 (0.012)	+0.005 (<0.001)	**+0.004 (0.005)**	**+0.004 (0.001)**
N = 1,001		Wald	Percentile	Bias-corrected	Wald	Percentile	Bias-corrected
vcov	CI90	+0.028 (0.018)	**–0.004 (0.003)**	–0.033 (0.015)	**+0.006 (0.001)**	+0.008 (0.001)	**+0.006 (0.002)**
	CI95	+0.018 (0.011)	**–0.006 (0.005)**	–0.020 (0.009)	**+0.002 (0.001)**	+0.004 (0.001)	**+0.002 (0.001)**
rvcov	CI90	+0.029 (0.014)	**–0.002 (0.007)**	–0.030 (0.015)	+0.013 (0.002)	+0.013 (0.003)	**+0.010 (0.004)**
	CI95	+0.017 (0.009)	**–0.006 (0.007)**	–0.019 (0.011)	+0.005 (0.001)	+0.005 (<0.001)	**+0.004 (0.002)**
N = 2,001		Wald	Percentile	Bias-corrected	Wald	Percentile	Bias-corrected
vcov	CI90	+0.019 (0.018)	**+0.004 (0.010)**	–0.018 (0.006)	**+0.002 (0.002)**	+0.003 (0.002)	**+0.002 (0.002)**
	CI95	+0.011 (0.011)	**+0.001 (0.005)**	–0.011 (0.003)	⋆ **(0.002)**	+0.001 (0.001)	⋆ **(0.002)**
rvcov	CI90	+0.023 (0.016)	**+0.008 (0.009)**	–0.015 (0.006)	+0.009 (0.002)	**+0.007 (0.002)**	**+0.007 (0.002)**
	CI95	+0.012 (0.010)	**+0.002 (0.005)**	–0.008 (0.003)	+0.004 (0.001)	+0.003 (0.001)	**+0.002 (0.002)**

Note. SDs between parentheses. For each population model, the CI method with the smallest deviation from ideal coverage per row is bolded.

⋆ Deviation between –0.001 and 0.001

Table S2: Deviation from ideal coverage, averaged over the 4 age values, for the 50th percentile

		SON-R 6-40			FEEST		
N = 501		Wald	Percentile	Bias-corrected	Wald	Percentile	Bias-corrected
vcov	CI90	**+0.003 (0.010)**	–0.012 (0.009)	–0.004 (0.010)	+0.003 (0.001)	**–0.002 (0.002)**	–0.004 (0.002)
	CI95	**+0.001 (0.006)**	–0.011 (0.005)	–0.006 (0.007)	**+0.001 (0.002)**	–0.006 (0.001)	–0.007 (0.002)
rvcov	CI90	+0.016 (0.013)	**–0.001 (0.010)**	–0.008 (0.012)	+0.016 (0.003)	**+0.012 (0.003)**	**+0.012 (0.002)**
	CI95	+0.007 (0.007)	–0.004 (0.007)	**–0.001 (0.008)**	+0.008 (0.003)	**+0.005 (0.002)**	**+0.005 (0.002)**
N = 1,001		Wald	Percentile	Bias-corrected	Wald	Percentile	Bias-corrected
vcov	CI90	**+0.001 (0.005)**	–0.007 (0.004)	–0.002 (0.005)	+0.005 (0.004)	⋆ **(0.003)**	–0.001 (0.003)
	CI95	**–0.001 (0.003)**	–0.007 (0.002)	–0.004 (0.003)	+0.004 (0.001)	–0.003 (0.001)	**–0.002 (0.002)**
rvcov	CI90	+0.009 (0.006)	**+0.001 (0.005)**	+0.006 (0.005)	+0.016 (0.003)	**+0.012 (0.002)**	**+0.012 (0.002)**
	CI95	+0.004 (0.004)	–0.002 (0.003)	**+0.001 (0.004)**	+0.008 (0.002)	**+0.005 (0.002)**	**+0.005 (0.001)**
N = 2,001		Wald	Percentile	Bias-corrected	Wald	Percentile	Bias-corrected
vcov	CI90	**–0.003 (0.002)**	–0.006 (0.003)	–0.008 (0.004)	+0.006 (0.003)	+0.003 (0.003)	**+0.001 (0.002)**
	CI95	**–0.003 (0.002)**	–0.005 (0.002)	–0.006 (0.002)	+0.004 (0.002)	**+0.002 (0.003)**	**+0.002 (0.003)**
rvcov	CI90	+0.003 (0.003)	**–0.001 (0.002)**	–0.007 (0.003)	+0.016 (0.001)	**+0.013 (0.002)**	+0.014 (0.002)
	CI95	⋆ **(0.003)**	–0.004 (0.001)	–0.005 (0.004)	+0.010 (0.003)	+0.008 (0.003)	**+0.004 (0.005)**

Note. SDs between parentheses. For each population model, the CI method with the smallest deviation from ideal coverage per row is bolded.

⋆ Deviation between –0.001 and 0.001

Table S3: Deviation from ideal coverage, averaged over the 4 age values, for the 95th percentile

		SON-R 6-40			FEEST		
N = 501		Wald	Percentile	Bias-corrected	Wald	Percentile	Bias-corrected
vcov	CI90	+0.020 (0.017)	**–0.015 (0.006)**	–0.018 (0.008)	**+0.014 (0.003)**	–0.025 (0.002)	–0.021 (0.002)
	CI95	**+0.012 (0.009)**	**–0.012 (0.003)**	–0.017 (0.005)	**–0.005 (0.004)**	–0.023 (0.002)	–0.020 (0.002)
rvcov	CI90	+0.016 (0.011)	**–0.013 (0.006)**	–0.018 (0.007)	+0.018 (0.002)	–0.005 (0.002)	**–0.003 (0.002)**
	CI95	**+0.006 (0.004)**	–0.016 (0.007)	–0.020 (0.009)	+0.012 (0.001)	–0.005 (0.002)	**–0.004 (0.001)**
N = 1,001		Wald	Percentile	Bias-corrected	Wald	Percentile	Bias-corrected
vcov	CI90	+0.012 (0.012)	**–0.006 (0.003)**	–0.010 (0.004)	+0.017 (0.003)	–0.007 (0.002)	**–0.004 (0.003)**
	CI95	**+0.007 (0.008)**	**–0.007 (0.001)**	–0.010 (0.003)	+0.010 (0.001)	–0.007 (0.001)	**–0.005 (0.001)**
rvcov	CI90	+0.016 (0.010)	**–0.006 (0.004)**	–0.010 (0.005)	+0.019 (0.002)	–0.004 (0.002)	**–0.003 (0.003)**
	CI95	**+0.008 (0.005)**	–0.009 (0.003)	–0.012 (0.005)	+0.012 (0.001)	–0.005 (0.001)	**–0.004 (0.001)**
N = 2,001		Wald	Percentile	Bias-corrected	Wald	Percentile	Bias-corrected
vcov	CI90	+0.006 (0.011)	**–0.003 (0.006)**	–0.004 (0.005)	+0.007 (0.005)	–0.002 (0.004)	**–0.001 (0.004)**
	CI95	+0.005 (0.006)	**–0.002 (0.004)**	–0.004 (0.003)	+0.005 (0.004)	**–0.002 (0.004)**	**–0.002 (0.003)**
rvcov	CI90	+0.005 (0.009)	**–0.003 (0.003)**	–0.005 (0.005)	+0.013 (0.005)	**+0.003 (0.005)**	–0.004 (0.006)
	CI95	**+0.003 (0.006)**	**–0.003 (0.001)**	–0.006 (0.001)	+0.009 (0.004)	⋆ **(0.004)**	+0.001 (0.004)

Note. SDs between parentheses. For each population model, the CI method with the smallest deviation from ideal coverage per row is bolded.

⋆ Deviation between –0.001 and 0.001
